# Effects of Personalized Mental Imagery Training on Anger Expression and Resilience in Adolescent Rugby Players: A Controlled Study

**DOI:** 10.3390/children13020249

**Published:** 2026-02-11

**Authors:** Donatella Di Corrado, Patrizia Tortella, Marinella Coco, Giuseppe Messina, Francesca Campoli, Maria Chiara Parisi

**Affiliations:** 1 Department of Sport Sciences, Kore University, Cittadella Universitaria, 94100 Enna, Italy; patrizia.tortella@unikore.it (P.T.); mariachiara.parisi@unikore.it (M.C.P.); 2 Department of Education Sciences, University of Catania, 95124 Catania, Italy; marinella.coco@unict.it; 3 Department of Human Sciences and Promotion of the Quality of Life, San Raffaele University, 20132 Rome, Italy; giuseppe.messina@uniroma5.it (G.M.); francesca.campoli@uniroma5.it (F.C.)

**Keywords:** mental imagery, resilience, anger expression, rugby, youth sport

## Abstract

**Highlights:**

**What are the main findings?**

- Personalized mental imagery training significantly improved imagery ability and resilience in adolescent rugby players.
- The intervention led to a meaningful reduction in maladaptive anger expression compared with an educational control condition.

**What is the implication of the main finding?**

- Mental imagery represents a feasible and low-cost strategy to support emotional regulation in high-contact youth sports.
- Strengthening resilience may be a key psychological mechanism through which imagery-based interventions reduce maladaptive anger responses.

**Abstract:**

**Background:** Adolescence is a critical developmental period marked by heightened emotional reactivity and increased exposure to stress, particularly in high-contact sports such as rugby. Maladaptive anger expression can negatively affect young athletes’ psychological well-being, behavior, and performance. Mental imagery may support emotional regulation by enabling athletes to rehearse adaptive cognitive and emotional responses. This study examined the effectiveness of a personalized mental imagery training program on imagery ability, resilience, and anger expression in adolescent rugby players and investigated whether resilience mediated the relationship between mental imagery and anger expression. **Methods:** A total of 120 male adolescent rugby players (mean age = 16.9 ± 2.01 years) were assigned to an experimental group (n = 62) or a time-matched educational control group (n = 58). **Results:** Mixed-design analyses of variance revealed significant Group × Time interactions for imagery ability, resilience, and anger expression, with medium-to-large effect sizes. Compared with the control group, the experimental group demonstrated greater improvements in imagery vividness and resilience, along with a significant reduction in maladaptive anger expression. Mediation analyses showed that resilience significantly mediated the relationship between mental imagery and anger expression, with full mediation for static imagery and partial mediation for dynamic imagery. **Conclusions:** Personalized mental imagery training effectively enhances emotional regulation in adolescent rugby players, primarily by strengthening resilience. Imagery-based interventions represent a feasible and effective approach to promoting adaptive emotional regulation and psychological well-being in high-contact youth sports.

## 1. Introduction

Adolescence represents a critical developmental period characterized by profound physical, cognitive, and emotional changes. During this stage, young athletes experience heightened emotional reactivity and are still developing effective strategies for emotional regulation and coping with stress [1]. In the context of competitive sport, these developmental challenges may be amplified by performance demands, social evaluation, and exposure to emotionally charged situations.

Rugby is a high-contact, open-skill team sport that places considerable physical and psychological demands on athletes. Adolescent rugby players are regularly exposed to intense physical collisions, competitive pressure, fatigue, and interpersonal confrontation. These factors create a context in which stress and anger-related responses are likely to emerge, particularly under conditions of physical exhaustion and perceived frustration [2]. Maladaptive emotional responses, such as poor anger regulation and impulsive reactions, may increase the likelihood of committing penalties during competition, thereby negatively affecting individual and team performance. [3].

Physical fatigue is known to impair cognitive control and emotional regulation, increasing vulnerability to stress and impulsive reactions. Accumulated fatigue has been shown to impair executive functioning and reduce cognitive control, thereby increasing vulnerability to impulsive emotional responses, including anger [4]. In adolescents, whose self-regulatory capacities are still developing, the interaction between fatigue and emotional stress may be particularly pronounced. Consequently, identifying psychological strategies that support anger expression management in physically demanding youth sports represents an important research priority.

Mental imagery is a widely used psychological skill in sport that involves the conscious simulation of movements, actions, and emotional experiences in the absence of physical execution [5]. Through imagery, athletes can mentally rehearse sport-specific skills, anticipate competitive demands, and prepare adaptive cognitive and emotional responses. A substantial body of research has demonstrated the effectiveness of imagery for enhancing performance, confidence, and psychological skills across a range of sports and age groups [6].

In youth and adolescent sport, mental imagery is considered particularly relevant due to ongoing cognitive and emotional development. Previous studies have shown that young athletes can use imagery effectively and that imagery ability can be enhanced through structured and guided practice [7,8]. Importantly, imagery-based interventions in youth sport have been associated not only with performance-related outcomes but also with improvements in psychological well-being and self-regulatory capacities [9]. However, much of the existing literature has focused on performance enhancement rather than emotional outcomes, leaving the role of imagery in emotional regulation, through its effects on anger expression, relatively underexplored.

Emotional regulation refers to the processes through which individuals influence the experience and expression of their emotions [10]. In sport, it is essential for maintaining attentional focus, behavioral control, and adaptive decision-making under pressure. Research has shown that mental imagery not only enhances athletic performance but also helps alleviate pre-competition stress symptoms [11]. Among the range of emotions experienced by athletes, anger is particularly salient in high-contact sports such as rugby, where provocation, physical confrontation, and perceived injustice are common [12]. While some level of controlled aggression may be functional in rugby, maladaptive anger expression can lead to negative behavioral and psychological consequences, especially in youth athletes.

Resilience has been identified as a key psychological resource that supports adaptation to stress and adversity in both general and sport-specific contexts. In adolescent athletes, resilience reflects the ability to cope effectively with pressure, recover from setbacks, and maintain psychological stability despite ongoing demands [13]. Empirical evidence suggests that higher levels of resilience are associated with better stress management, lower emotional reactivity, and more adaptive coping strategies in sport [14].

Importantly, resilience is increasingly conceptualized as a dynamic and developable capacity rather than a fixed personality trait. Psychological interventions targeting cognitive appraisal, coping skills, and emotional awareness have been shown to enhance resilience in youth populations [15]. Within high-contact sports such as rugby, resilience may play a particularly important role in buffering the emotional impact of fatigue, physical contact, and competitive stress. Resilient athletes are better equipped to tolerate frustration, regulate anger, and recover quickly from emotionally challenging situations.

Recent research has begun to explore the relationship between mental imagery and resilience in sport [16].

Imagery may contribute to resilience by allowing athletes to mentally rehearse stressful scenarios and practice adaptive responses in a controlled environment. Through repeated exposure to simulated adversity, athletes may develop emotional preparedness, perceived control, and tolerance to stress—core components of resilience [17]. Studies conducted with adult and student athletes have reported positive associations between imagery use and resilience-related constructs, such as confidence and coping effectiveness [18].

Despite the theoretical relevance of these constructs, limited empirical research has examined the relationships among mental imagery, resilience, and anger expression in rugby athletes. Moreover, few studies have tested these relationships within the context of a structured psychological intervention while controlling for the demanding physical and emotional load of regular rugby training. Addressing these gaps is essential for advancing both theory and practice in youth sport psychology.

### Study Aims and Hypotheses

The present study aimed to examine the psychological effects of personalized mental imagery training on emotional regulation in adolescent rugby players. Given the high physical demands and emotionally challenging nature of rugby, this study focused specifically on anger expression as a key emotional outcome.

A second aim was to investigate the role of resilience as a psychological mechanism linking mental imagery abilities to anger expression. Resilience was conceptualized as a dynamic resource that supports adolescents’ capacity to cope with stress, tolerate frustration, and regulate emotions in demanding sport contexts.

In addition, this study evaluated the effectiveness of a structured mental imagery training program delivered alongside regular rugby training, compared with a time-matched educational control condition. It was hypothesized that athletes in the experimental group would show greater improvements in imagery vividness and resilience, along with greater reductions in maladaptive anger expression, than those in the control group. Furthermore, resilience was expected to mediate the relationship between mental imagery and anger expression. By integrating theoretical and applied perspectives, this research contributes to a deeper understanding of how psychological skills training can promote emotional well-being, resilience, and adaptive functioning in adolescent athletes participating in high-contact sports.

## 2. Materials and Methods

### 2.1. Sample Size Calculation and Participants

A priori power analysis was conducted using G*Power (version 3.1.9.7) [19] to estimate the required sample size. This analysis found a multiple regression analysis with 5 predictors, an alpha level of 0.05, 80% power, and an effect size of f^2^ = 0.15, with a required total sample size of N = 120. Therefore, a total of n = 123 participants were enrolled in this study.

All athletes were recruited from local rugby clubs competing at a regional level and had a minimum of three years of structured rugby training experience. Inclusion criteria were: (a) current participation in organized rugby training and competition, (b) absence of self-reported neurological or psychiatric disorders, and (c) no previous participation in formal psychological skills training programs focused on mental imagery during the six months preceding this study. After exclusions due to injury (n = 3), the final analytical sample consisted of 120 male competitive rugby players aged between 15 and 18 years old (mean age = 16.9 years; ± 2.01). They were randomly assigned to an experimental group (n = 62) or a control group (n = 58) using a computer-generated randomization sequence. Minor differences in group size reflect the random allocation process.

Throughout this study, all participants continued their regular rugby training routines, which consisted of three to four on-field sessions per week (90–120 min per session) and one competitive match per week. Training sessions included technical skill development (e.g., passing, tackling, ruck and scrum drills), tactical exercises (e.g., team organization and decision-making under pressure), and physical conditioning (e.g., strength, speed, and endurance work). These training demands typically induce high levels of physical fatigue, competitive stress, and emotionally challenging situations, such as repeated physical contact, performance errors, and interpersonal confrontation.

All participants received a detailed explanation of the study procedures prior to their initiation. Written informed consent was obtained from their parents or legal guardians before any testing was conducted. This study was carried out in compliance with the Ethical Code of the University of Palermo and the Code of Ethics approved by the General Assembly of the Italian Association of Psychology on 27 March 2015. All procedures adhered to the principles outlined in the Declaration of Helsinki, and ethical approval was granted by the University Enna Kore Internal Review Board (UKE-IRBPSY-05.25.11).

### 2.2. Procedures

#### 2.2.1. Assessment

Measures of mental imagery, resilience, and anger expression were collected before T_0_ (baseline) and after the intervention period (T_1_). The assessment took place in a quiet and secluded area within the training facilities with analogous conditions (room temperature 21 °C, electric illumination, and time of day) under the supervision of two researchers, and the confidentiality of their responses was guaranteed. During the assessment sessions, participants were not allowed to communicate, and no feedback was provided.

#### 2.2.2. Intervention

##### Experimental Group: Personalized Mental Imagery Training

Participants assigned to the experimental group completed a personalized mental imagery training program designed to enhance resilience and emotional regulation in rugby-specific contexts. The intervention lasted four weeks and consisted of three sessions per week, with each session lasting approximately 20 min (12 sessions in total). The training was delivered in addition to regular rugby training.

Each session followed a standardized structure including: (a) brief emotional check-in; (b) centring and breathing exercises; (c) guided multi-sensory imagery using first-person perspective; (d) rehearsal of rugby-specific scenarios involving physical contact, fatigue, and emotional provocation; (e) practice of adaptive coping responses; and (f) debriefing and reinforcement of personal cue words.

Imagery scenarios were individualized based on athletes’ self-reported anger triggers, baseline imagery ability, and playing position. Progressive exposure to emotionally demanding situations was implemented across the intervention period, with increasing intensity of simulated stressors and emphasis on rapid emotional recovery (“bounce-back”) strategies.

##### Control Group: Standardized Imagery Education

Participants assigned to the control group received a time-matched standardized educational program focused on mental imagery and mental training in sport. The program consisted of three sessions per week over four weeks, with each session lasting approximately 20 min.

Sessions included reading materials and video-based content introducing mental imagery concepts, imagery modalities, and general applications in sport psychology. No guided imagery practice, emotional rehearsal, or individualized feedback was provided. This condition was designed to control exposure to imagery-related information while minimizing active psychological skill development.

### 2.3. Measurements

#### 2.3.1. Imagery Vividness Assessments

##### Static Imagery

Static imagery was assessed using the Vividness of Visual Imagery Questionnaire (VVIQ) [20], in its Italian adapted version [21]. The VVIQ is a 16-item self-report instrument comprising four subscales (four items each) designed to evaluate the vividness of visual mental imagery (e.g., imagining the appearance of a friend or parent, climatic conditions, or landscapes). After imagining each scene, participants rated the clarity and vividness of their mental images on a five-point scale ranging from 1 (“No image at all, only knowing that you are thinking of the object”) to 5 (“Perfectly clear and vivid as normal”). Higher scores indicate greater imagery vividness. The questionnaire demonstrated good internal consistency, with an Omega coefficient (Ω) of 0.85, and high test–retest reliability (r = 0.89) [22].

##### Dynamic Imagery

Dynamic imagery was measured using the Vividness of Movement Imagery Questionnaire–2 (VMIQ-2) [23], a 12-item self-report questionnaire assessing the ability to imagine movements both visually and kinesthetically. Responses are provided on a five-point Likert scale ranging from 1 (“Perfectly clear and vivid”) to 5 (“No image at all; you only know that you are thinking of the movement”). VMIQ-2 evaluates three types of movement imagery: (1) external dynamic imagery, referring to observing oneself performing a movement from an external perspective; (2) internal dynamic imagery, involving viewing the movement from a first-person perspective; and (3) kinesthetic dynamic imagery, which reflects the sensation of performing the movement, as if executing the task. In the present study, the scale showed very good internal consistency (Ω = 0.81) and strong test–retest reliability (r = 0.87).

##### State-Trait Anger Expression Inventory-2 (STAXI-2)

The STAXI-2 [24] is a questionnaire consisting of 57 items that assess the expression of anger intended as an indicator of emotional regulation (anger/out, anger/in, control/out, control/in item, e.g., “I lose my temper”; “I control my temper”; “I express my anger”; “I take a deep breath and relax”). Ratings are on a 4-point frequency scale, from (1) Almost never to (4) Almost always. The STAXI-2 exhibits good reliability, with alpha coefficients ranging from 0.81 to 0.93. An index of anger expression can be derived to provide a summarizing measure of the expression and control of anger. In this study, we used the Italian adaptation of STAXI-2 [25]. We reported the internal consistency for the current study (Ω = 0.80) and a strong test-retest reliability (r = 0.83).

##### Resilience

The Resilience Scale [26] in the Italian adapted version [27] was used to evaluate the level of resilience. It is a 10-item questionnaire measuring resilience as the capacity to resist the effects of life stressors, thrive on challenges and promote adaptation (e.g., “I usually manage one way or another”; “I am determined”; “My life has meaning”; “When I am in a difficult situation, I can usually find a solution”). Response options range from 1 (“strongly disagree”) to 7 (“strongly agree”), with higher scores reflecting higher levels of resilience. The Scale exhibits good reliability, with alpha coefficients ranging from 0.70 to 0.91. The Italian adaptation confirmed good psychometric properties and provided support for the construct validity of the scale. In the current sample, the Omega coefficient (Ω) for scale was 0.81, with a test-retest reliability correlation of 0.83.

### 2.4. Statistical Analysis

Statistical analyses were performed using SPSS (version 27.0; IBM Corp., Armonk, NY, USA). Descriptive statistics (means and standard deviations) were calculated for all variables. Baseline differences between the experimental group (n = 62) and the control group (n = 58) were examined using independent-samples *t* tests for continuous variables.

Intervention effects were analysed using mixed-design analyses of variance (ANOVA), with Group (experimental vs. control) as the between-subjects factor and Time (pre-intervention vs. post-intervention) as the within-subjects factor for each outcome measure (mental imagery, resilience, and anger expression). Effect sizes were estimated using partial eta-squared (η^2^p) and interpreted according to conventional benchmarks, with values of 0.01, 0.06, and 0.14 representing small, medium, and large effects, respectively.

Furthermore, Pearson’s correlation was used to determine the relationships between the selected variables. To examine the significance of mediation, the recommendations put forth by Baron and Kenny [28] were followed. Their Steps 1, 2 and 3 involve testing the significance of the relationship between the following: (1) the independent and the dependent variables (i.e., imagery and anger); (2) the independent and the mediator variables (i.e., imagery and resilience); (3) the mediator and the dependent variables (i.e., resilience and anger). If these steps are passed, one should determine in Step 4 whether the mediator variable reduces or eliminates the link between the independent variable and the dependent variable. For this purpose, a mediation analysis was conducted using Hayes’ [29] PROCESS version 3.1 (Hayes PROCESS macro-Model 4—Ohio, USA) computational tool for SPSS. This tool enables the estimation of path coefficients, standard errors, and different indexes of effect size, as well as the significance of the indirect effects obtained through the bootstrapping method with 5000 repetitions, with a confidence interval (CI) of 95% [30]. Statistical significance was set at *p* ≤ 0.05.

## 3. Results

Anthropometric characteristics of participants are presented in Table 1. No significant differences were observed between the experimental and control groups in age, height, or weight (*p* > 0.05), indicating baseline equivalence.

To examine the effects of the intervention, mixed-design ANOVAs were conducted with Group (experimental vs. control) as the between-subject factor and Time (pre-intervention vs. post-intervention) as the within-subject factor for each outcome variable (Table 2).

Significant Group × Time interactions were observed for resilience; static imagery; external, internal, and kinesthetic dynamic imagery; and anger expression, indicating differential changes over time between the experimental and control groups.

Post-hoc analyses showed that participants in the experimental group demonstrated significant improvements in resilience and imagery abilities from pre- to post-intervention, accompanied by a significant reduction in maladaptive anger expression. In contrast, the control group exhibited only small changes across variables. Effect sizes for the interaction effects were medium to large (η^2^p = 0.14–0.23), supporting the practical relevance of the intervention.

Pearson correlation analyses (Table 3) revealed that all imagery dimensions were significantly and positively intercorrelated (r = 0.35–0.67, *p* < 0.001). Resilience was positively associated with all imagery dimensions (r = 0.61–0.64, *p* < 0.001), whereas anger expression was negatively correlated with imagery abilities (r = −0.62 to −0.69, *p* < 0.001) and resilience (r = −0.65, *p* < 0.001). These findings indicate that athletes with stronger imagery abilities and higher resilience reported lower levels of maladaptive anger expression.

### Mediation Analysis

Mediation analyses (Table 4) indicated that resilience significantly mediated the relationship between mental imagery dimensions and anger expression. For static imagery, the indirect effect through resilience was significant while the direct effect was not, indicating full mediation. For external, internal, and kinesthetic dynamic imagery, both indirect and direct effects remained significant, suggesting partial mediation.

## 4. Discussion

The present study examined the psychological mechanisms underlying anger expression in adolescent rugby athletes by investigating the mediating role of resilience in the relationship between mental imagery and anger expression. In addition, this study evaluated the effectiveness of a personalized mental imagery training program delivered alongside regular rugby training. Overall, the findings support the study hypotheses, showing that imagery training was associated with improvements in imagery ability and resilience, as well as reductions in maladaptive anger expression. Importantly, resilience emerged as a key psychological mechanism linking mental imagery to anger expression [31].

Athletes who participated in the personalized mental imagery training demonstrated greater improvements in imagery ability compared to the control group. This finding highlights the importance of structured, guided, and individualized imagery practice [32,33]. While the control group received educational information about imagery, the absence of active rehearsal, emotional simulation, and personalized feedback likely limited the development of functional imagery skills.

Beyond imagery ability, the intervention produced meaningful increases in resilience. Athletes in the experimental group showed substantial gains in resilience over time, whereas resilience remained relatively stable in the control group. This result supports the view that resilience is a developable psychological capacity rather than a fixed trait [34]. Through repeated mental simulation of challenging rugby-specific situations and rehearsal of adaptive coping responses, athletes may strengthen their ability to tolerate stress, recover from setbacks, and maintain emotional stability [35].

A central finding of this study is the reduction in maladaptive anger observed in the experimental group. Rugby is characterized by frequent physical contact, high physiological arousal, and emotionally provocative situations, particularly under conditions of fatigue [36]. Poor anger expression in this context may lead to impulsive behaviors, penalties, and impaired performance. The observed reduction in maladaptive anger expression suggests that imagery training may develop athletes’ capacity to regulate emotional responses during demanding situations [37].

The correlational and mediation analyses provide further insight into the mechanisms underlying these effects. Imagery abilities were positively associated with resilience and negatively associated with anger expression, while resilience showed a strong inverse relationship with anger. Importantly, resilience fully mediated the relationship between static imagery and anger expression, indicating that the effects of static imagery on anger operate primarily through enhanced resilience. In contrast, dynamic imagery modalities showed partial mediation, suggesting that they may influence anger expression both directly and indirectly through resilience.

This distinction underscores the functional relevance of different imagery components. Static imagery may primarily support emotional regulation by strengthening coping resources and emotional stability, whereas dynamic imagery—particularly kinesthetic imagery—may exert additional direct effects by enhancing embodied awareness, emotional simulation, and self-regulatory control during action [38].

From an applied perspective, identifying resilience as a mechanism has important implications for psychological interventions in high-contact sports. Imagery-based training programs that explicitly target emotional preparation, coping, and recovery may be particularly effective in fostering resilience and reducing maladaptive anger responses [39].

A key strength of the present study is the integration of psychological intervention within regular rugby training. Athletes continued to experience high physical loads, fatigue, and emotionally demanding situations, enhancing ecological validity. The observed psychological improvements under these conditions suggest that imagery training may function as a protective resource, supporting emotional regulation despite ongoing physical and competitive stress [40].

### Limitations and Future Directions

Despite its strengths, this study has several limitations. First, psychological constructs were assessed using self-report measures, which may be subject to response bias. Moreover, although emotional regulation was the overarching construct of interest, it was operationalized in this study through self-reported measures of anger expression. Future research should include behavioral and physiological indicators to capture emotional regulation more comprehensively. Second, although the intervention demonstrated short-term effects, the durability of these changes over time remains unknown. Longitudinal follow-up assessments would help determine the long-term impact of imagery training on resilience and emotional regulation.

Future research could also examine whether similar mechanisms operate in other high-contact or endurance sports and explore potential moderating variables, such as training load, playing position, and competitive level. Additionally, integrating physiological markers of fatigue or stress may further clarify the interaction between physical demands and psychological regulation.

Finally, an important limitation of the present study is the absence of direct observational measures of maladaptive anger-related behaviors during match play. Although in-game behavioral indicators could provide greater ecological validity, substantial variability in match exposure, playing time, and competitive context across participants, together with the lack of standardized behavioral statistics across teams, limited the feasibility of reliable pre–post observational assessment. Future longitudinal studies should incorporate systematic behavioral coding of anger expressions across multiple competitive matches to complement self-report measures and further clarify the impact of mental imagery interventions on in-game behavior.

## 5. Conclusions

The present study provides evidence that resilience mediates the relationship between mental imagery and anger expression in adolescent rugby players. A personalized mental imagery training program delivered alongside regular rugby training was associated with greater improvements in imagery ability and resilience, as well as a more adaptive pattern of anger expression, compared with an educational control condition.

These findings contribute to a clearer understanding of emotional regulation in high-contact sports, indicating that improvements in emotion regulation may be observed through reductions in maladaptive anger expression. Resilience emerged as a key psychological mechanism through which mental imagery exerts its effects, highlighting its central role in coping with physical fatigue, competitive stress, and emotionally provocative situations. From an applied perspective, imagery-based interventions specifically designed to simulate emotionally demanding sport scenarios and rehearse adaptive coping responses may represent an effective strategy for enhancing resilience and managing anger expression in youth sport environments. Integrating such interventions into regular training programs may support both psychological well-being and adaptive performance in adolescent athletes.

## Figures and Tables

**Table 1:** Participants’ anthropometric characteristics (M ± SD).

	Age [Years]	Height [m]	Weight [kg]
EG	16.9 ± 1.9	1.74 ± 0.06	84.7 ± 7.29
CG	16.9 ± 1.7	1.76 ± 0.05	87.8 ± 6.32
*p*	0.771	0.691	0.682

**Note.** EG is the experimental group and CG is the control group.

**Table 2:** Pre- and post-intervention comparisons between experimental and control groups.

Variables	Group	Pre-T_0_	Post-T_1_	Δ Post-Pre	*p*	F (Group × Time)	η^2^p
Resilience	Experimental	55.06 ± 3.11	65.84 ± 2.56	10.78 ± 3.48 *	0.001	31.06	0.19
	Control	51.41 ± 2.39	53.32 ± 2.26	1.91 ± 1.32			
Static Imagery	Experimental	43.51 ± 4.17	68.32 ± 2.43	16.81 ± 5.48 *	0.001	41.16	0.23
	Control	41.18 ± 4.02	46.01 ± 3.85	4.83 ± 4.08			
External dynamic Imagery	Experimental	25.32 ± 2.57	48.01 ± 6.03	22.69 ± 3.28 *	0.001	21.98	0.14
	Control	23.62 ± 3.49	28.63 ± 3.03	5.01 ± 1.42			
Internal dynamic Imagery	Experimental	28.12 ± 6.25	42.95 ± 6.75	14.83 ± 2.11 *	0.001	22.06	0.15
	Control	29.91 ± 6.04	30.6 ± 6.01	0.69 ± 0.78			
Kinesthetic dynamic Imagery	Experimental	32.12 ± 3.60	50.03 ± 5.24	17.91 ± 4.48 *	0.001	20.58	0.14
	Control	31.45 ± 3.21	39.11 ± 4.03	7.66 ± 3.42			
Anger	Experimental	61.21 ± 5.60	49.22 ± 4.24	−11.99 ± 3.48 *	0.001	31.98	0.15
	Control	62.34 ± 5.21	54.45 ± 4.18	−7.89 ± 0.42			

**Note.** * *p* < 0.001. Values are expressed as mean ± standard deviation. F values refer to the Group × Time interaction from mixed-design ANOVA. η^2^p = partial eta squared.

**Table 3:** Pearson’s correlation coefficients.

Variable	1	2	3	4	5	6
1. Static Imagery	1					
2. External dynamic Imagery	0.67 *	1				
3. Internal dynamic Imagery	0.64 *	0.63 *	1			
4. Kinesthetic dynamic Imagery	0.41 *	0.35 *	0.44 *	1		
5. Resilience	0.64 *	0.62 *	0.61 *	0.63 *	1	
6. Anger	−0.69 *	−0.67 *	−0.62 *	−0.63 *	−0.65 *	1

* *p* < 0.001.

**Table 4:** Effects of imagery on anger expression through resilience (standardized β).

Paths	Indirect Effect			Direct Effect		
	β	CI. 95%	*p*	β	CI. 95%	*p*
Static Imagery->Resilience->Anger	1.04	[0.72, 1.36]	<0.001	0.24	[−0.13, 0.58]	0.20
External dynamic Imagery->Resilience->Anger	−0.57	[−0.84, −0.37]	<0.001	−0.92	[−1.17, −0.66]	<0.001
Internal dynamic Imagery->Resilience->Anger	−0.81	[−1.03, –0.59]	<0.001	−0.66	[−0.83, −0.38]	<0.001
kinesthetic dynamic Imagery->Resilience->Anger	−0.46	[−0.68, −0.27]	<0.001	−0.54	[−0.75, −0.32]	<0.001

## Data Availability

The data that support the findings of this study are available from the corresponding author (D.D.C.), upon reasonable request.
